# Medical History from the Earliest Times

**Published:** 1892-05-28

**Authors:** E. T. Withington


### Medical History from the Earliest Times.

By E. T. Withington, M.A., M.B. Oxon.

### X.—HIPPOCRATES.

The remark wittily made on the Homeric question, that the poems were not written by Homer, but by another man of the same name, might probably be applied with literal truth to some of the Hippocratic writings, for we know no less than seven physicians named Hippocrates, who lived during the period when this collection was being formed. It is the second of these, the probable author of about one-fourth of the treatises, who is the most famous of all physicians, and of whom some account must now be attempted. Born B.C. 460, the supposed descendant of Æsculapius and Hercules, Hippocrates lived during that wonderful epoch called the age of Pericles, when there appeared, within the narrow limits of the Greek world, more men of genius than have, perhaps, existed together in Europe since. Biographical details do not come within our present scope, and the biography of Hippocrates is almost entirely apocryphal. He is universally known as the Father of Medicine. He was called "The Great" even by his own contemporaries. In the darkest ages of medical ignorance the term "Hippocratic" still denoted all that was considered best and highest in the art. At the Rénaissance, the wildest revolutionists, who despised Galen and Avicenna, still reverenced Hippocrates, and his name has been inscribed upon the banners of all the reformers of medicine from the sixteenth century downwards. It will be well, therefore, before attempting an account of the Hippocratic medicine, to discuss a few of those services which justify the pre-eminence thus given to the great Asclepiad.

Though there were physicians before Hippocrates, to whose discoveries he himself always renders due honour, the reforms he introduced were so important, and the impress which his genius left upon the whole art was so great, that the title, Father of Medicine, is well deserved. Three only of the principal aspects of his work can be here discussed; his introduction of more detailed observations of disease, the high importance which he attributed to prognosis, and his rejection of the supernatural in medicine.

Hippocrates has left us forty-two clinical histories, almost the only ones which have come down from antiquity, and of these the shortest may be taken as an example, which should be compared, or, rather, contrasted with the temple inscription given above.

"Seventh case (of the second series).—A woman at the house of Aristion with sore throat, which began from the tongue; speech indistinct, tongue red, and became parched. First day, she felt chilly, and was then feverish. Third day, a rigor and acute fever; a reddish, hard œdema on both sides of the neck and chest; extremities cold and livid; respiration laboured; fluids returned through the nose; could not drink; constipation and suppression of urine. Fourth day, all the symptoms grew worse. Fifth day, the patient died." Neither here nor in any other case does Hippocrates name the disease, giving opportunity for many diagnostic attempts by the commentators. Thus Wunderlich suggests that the above was a case of scarlatina, with acute nephritis, while others have called it erysipelas. With the exception of a few cases in which bleeding or enemata are mentioned, there is no notice of the remedies employed, perhaps because the ordinary treatment of such cases was generally known. No less than twenty-five of the forty-two cases end fatally, a fact brought forward by all the commentators as an instance of the scientific honesty of Hippocrates, and of that dislike of anything approaching display or quackery which is seen in all his writings.

The taking of clinical histories, of which the above is an embryonic type, is now justly considered one of the most important parts of a medical education, but it is an amazing fact that, with one or two exceptions, the example thus given by Hippocrates was not followed for nearly two thousand years, and it was the revival of the practice, especially by our own Sydenham (upon whom, as we shall see, the two treatises containing these cases made a deep impression) that did most to inaugurate the clinical medicine of modern times. Still there can be no doubt that at all times the students of these writings must have had their minds directed to the importance of detailed observations of disease, and it is probably to this doctrine that the art of healing depends, not on theory, but on observation, that Celsus refers when he says that Hippocrates first separated medicine from philosophy.

But what chiefly strikes a modern reader is the importance attributed to prognosis. The wish to know the future was strongly characteristic of the Greek mind, as is seen in their numerous oracles, and Æschylus makes the chorus in the "Prometheus" say, "It is pleasant for the sick to know clearly beforehand what pain is to come." Hippocrates, indeed, admits that it is better to cure the patient than to tell him what is going to happen; but this, he says, is not always possible, and failing it, he is the best physician who can give the most correct prognosis. The Hippocratic prognosis, however, is more than mere prophecy, it includes the entire natural history of the disease (in the literal meaning of those words), considered as a whole, and in its relation to the patient's organism. This, we must admit, is a fundamental part of medicine, for it is clearly impossible to judge the value of any treatment unless we know the natural tendencies of the disorder. And just as the revival of Hippocratic observation in the seventeenth century gave a new birth to clinical medicine, so the revival of the Hippocratic prognosis in our own days—though tending at one time to produce an exaggerated expectancy, not to say nihilism in treatment—had no small share in bringing about the more modern revolutions in medical practice.

It has been well said that great men illuminate the world by gathering into a focus the rays emanating from itself, and this is well seen in Hippocrates' third great service to medicine—his rejection of supernatural theories of disease. The age was one of transition, and the simple faith in the old mythology was giving way in all directions. Thus even the pious Herodotus ascribes the plague which fell upon the retreating Persians, not to the avenging gods of Hellas, but to the famine and hardships which they underwent; and though he considers the madness of Cleomenes an appropriate punishment for his crimes, he thinks it may have been more directly due to a habit of hard drinking, acquired among the Scythians. The famous treatise on the "sacred disease"—epilepsy—which most clearly asserts the natural origin of sickness, is not certainly genuine, but in an undoubtedly Hippocratic work, the "Airs, Waters, and Places," we find the assertions that "no one disease is either more divine or more human than another," and that "none arises without a natural cause." The importance of this doctrine will be more apparent when we come to describe the disastrous effects upon medical progress produced by the revival of the old theories.

Space does not permit the consideration of the "Airs, Waters, and Places" a work in which Hippocrates founded not only historical and geographical medicine, but the philosophy of history generally; or of those famous Aphorisms which were for ages classed among the most wonderful products of human genius, and the majestic introduction to which would alone suffice to immortalise its author. But the personal character of Hippocrates must not be entirely passed over. No one ever had a highersense of the dignity of medicine; none showed greater respect for his patients; he even warns his pupils against exposing them unnecessarily during examination, or whilst operating. The great object of the physician should be to benefit his patient, or at least do him no harm, a sentiment which Galen thought at first unworthy of the Master, till he learnt its value from experience. The wishes, and even the whims of the patient are to be indulged as far as possible, and a physician should rather lose his fee than trouble a sick person about it, for the memory of a good deed is better than a temporary advantage. He should also neglect no opportunity of serving the poor and the stranger, for "where the love of the art is, there is the love of man." This last quotation, indeed, is from a work of very doubtful authorship, but it expresses the spirit, if not the words of Hippocrates.

It has been suggested that, when Aristophanes mentions physicians among those who have written about the "Clouds," he is referring to the "Airs, Waters, and Places," and that the epithet he there uses, "lazy long-haired fops with their rings and natty nails," is an attack on the personal appearance of Hippocrates himself; if so, the Father of Medicine need not have been ashamed to be included in a satire directed against the Father of Philosophy. But we may more appropriately conclude with the words of another poet, just twenty years older than the physician, who may have been thinking of Hippocrates when he wrote—

> "Happy the man who studies Nature's lore!
> Him neither evil thoughts can e'er entice,
> Nor party strife of angry citizens;
> But, pure in heart and hand, he scans the face
> Of her the Immortal Mother ever young."
>
> —*Euripides*, fragment.

